# Mediating effects of artificial intelligence on the relationship between academic engagement and mental health among Chinese college students

**DOI:** 10.3389/fpsyg.2024.1477470

**Published:** 2024-11-07

**Authors:** Yalin Wang, Hui Wang

**Affiliations:** ^1^Department of Marxism, Zhoukou Vocational and Technical College, Zhoukou, China; ^2^Department of Plant Protection, Henan Agricultural University, Zhengzhou, China

**Keywords:** artificial intelligence, mental health, theory of planned behavior, attitudes, subjective norm, behavioral intentions, perceived behavioral control, academic engagement

## Abstract

**Introduction:**

Academic engagement of Chinese college students has received increasing research attention due to its impact on Students’ Mental health and wellbeing. The emergence of artificial intelligence (AI) technologies marked the beginning of a new era in education, offering innovative tools and approaches to enhance learning. Still, it can be viewed from positive and negative perspectives. This study utilizes the Theory of Planned Behavior (TPB) as a theoretical framework to analyze the mediating role of students’ attitudes toward AI, perceived social norms, perceived behavioral control, and their intention to use AI technologies in the relationships between Students’ academic engagement and Mental health.

**Methods:**

The study involved a total of 2,423 Chinese college students with a mean age of approximately 20.53 ± 1.51 years. The survey was conducted through Questionnaire Star, using a secure website designed specifically for the study. The Hayes’ PROCESS Macro (Version 4.2) Model 80 with SPSS 29.0, a multivariate regression analysis with a chain mediation model that allows for multiple mediators to be tested sequentially, has been used. The statistical test explored the direct and indirect effects of students’ engagement (X) on mental health (Y) through a series of mediators: attitude toward AI (M1), subjective norm (M2), perceived behavioral control over AI use (M3), and AI use behavioral intention (M4).

**Results:**

The direct positive relationship between engagement and mental health (β = 0.0575; *p* < 0.05), as well as identifying key mediating factors such as perceived behavioral control (β = 0.1039; *p* < 0.05) and AI use of behavioral intention (β = 0.0672; *p* < 0.05), highlights the potential of AI tools in enhancing students’ well-being. However, the non-significant mediating effects of attitude toward AI (β = 0.0135), and subjective norms (β = –0.0005), suggest that more research is needed to understand the nuances of these relationships fully.

**Discussion:**

Overall, the study contributes to the growing body of literature on the role of AI in education and offers practical implications for improving mental health support in academic settings.

## Introduction

The relationship between academic engagement and mental health has emerged as a critical area of research, particularly among college students, who often face significant academic and social pressures. Academic engagement refers to the level of interest, participation, and commitment that students demonstrate toward their studies, and it includes four key dimensions: affective, social, cognitive, and behavioral (Bowden, 2022; Bowden et al., 2021). These dimensions shape not only the academic experience but also student well-being. For Chinese college students, the importance of academic engagement is especially pronounced due to unique cultural and educational expectations. Research has consistently shown that high levels of academic engagement are associated with positive mental health outcomes, while disengagement can lead to stress, anxiety, and depression (Sinval et al., 2024; Tang and He, 2023).

Increased socioeconomic development and supportive national educational policies in China have played an important role in enhancing academic engagement, which in turn has positively influenced student mental health. For instance, Li F. et al. (2022) reported significant improvements in mental health among Chinese physical education college students from 1995 to 2019, attributing this improvement to rising levels of engagement fostered by socioeconomic growth and policy interventions. Despite the growing body of research on this topic, there remains a need for more empirical studies, particularly to explore the cross-cultural and context-specific impacts of academic engagement and stress on student mental health (Arterberry et al., 2024; Beroiza-Valenzuela, 2024; Dave et al., 2024).

In recent years, the integration of artificial intelligence (AI) in higher education has transformed both learning and teaching environments, particularly in the realm of mental health education (Li Z., 2023). This integration can be understood from two perspectives: the use of AI at the organizational level, where institutions and teachers implement AI-based interventions, and the use of AI by students in their own learning activities. From the organizational standpoint, AI has been instrumental in improving the effectiveness of mental health education through personalized, scalable, and accessible interventions, including cognitive-behavioral approaches that focus on enhancing students’ psychological well-being (Aminah et al., 2023; Wang et al., 2024). Wang’s et al. (2024) cognitive-behavioral model of psychological education, guided by AI, is one such example, which significantly increased student engagement and participation in mental health activities.

On the student side, the adoption of AI tools for learning activities has been explored through frameworks such as the Unified Theory of Acceptance and Use of Technology (UTAUT). This model emphasizes the importance of factors such as performance expectancy, effort expectancy, and social influence in shaping students’ use of AI technologies (Venkatesh et al., 2012). Recent research has suggested that AI adoption by students can improve their academic engagement, helping them cope with academic stress and enhance their mental health (Alqahtani et al., 2023; Zapata et al., 2024). However, criticisms of the UTAUT model have pointed out its limitations in addressing the social ramifications of new technologies and the complexities of AI adoption in educational contexts (Venkatesh et al., 2012). Additionally, the integration of AI in classrooms—such as in flipped classroom models—has been shown to boost engagement and improve student performance in mental health courses (Shan and Liu, 2021). While these benefits are clear, there are still concerns regarding the ethical implications of AI, including privacy and data security issues, as well as the risk of diminishing human interaction (Escotet, 2023).

These complexities can be understood through the theoretical framework of the Theory of Planned Behavior (TPB). According to TPB, behavior is influenced by three components: attitudes toward the behavior, subjective norms, and perceived behavioral control (Ajzen, 1991). In this context, positive attitudes toward AI in education, supportive social norms, and students’ confidence in using AI tools can significantly influence their intention to adopt AI. For example, attitudes toward AI have been shown to predict behavior in academic-related fields, including help-seeking (Bornschlegl et al., 2021), cheating intentions (Stone et al., 2010), and AI use among both teachers and students (Abdelmoneim et al., 2024; Duong et al., 2024). Studies have demonstrated that positive attitudes, favorable subjective norms, and high perceived control are associated with higher engagement with AI tools, which can improve both academic outcomes and mental health (Wang et al., 2024; You and Zhang, 2024).

Additionally, perceived behavioral control plays a significant role in determining whether students feel capable of using AI technologies effectively. Factors such as access to user-friendly applications, institutional support, and training programs can increase perceived behavioral control and, subsequently, AI adoption (Li X. et al., 2022; Shan and Liu, 2021). A supportive environment that addresses students’ attitudes, subjective norms, and perceived control can help foster positive behavioral intentions toward AI use, which may lead to improved academic engagement and mental health outcomes.

This study aims to explore how attitudes toward AI, subjective norms, perceived behavioral control, and behavioral intention influence the relationship between Chinese college students’ academic engagement and mental health. By integrating the Theory of Planned Behavior into this framework, the study seeks to answer the following research question: How do attitudes toward AI, subjective norms, perceived behavioral control, and AI use behavioral intention influence the relationship between Chinese college students’ academic engagement and their mental health?

By addressing this question, we aim to provide insights into how AI adoption can enhance the learning experience and support mental well-being among Chinese college students. The findings from this study will not only contribute to the academic understanding of AI’s role in higher education but also inform the development of effective policies and interventions. These could help leverage AI technologies to create more personalized, supportive, and engaging learning environments that promote holistic student development. Furthermore, the research has the potential to guide educational institutions in promoting the effective use of AI to foster active learning, increase academic engagement, and improve student mental health.

**Figure 1:**
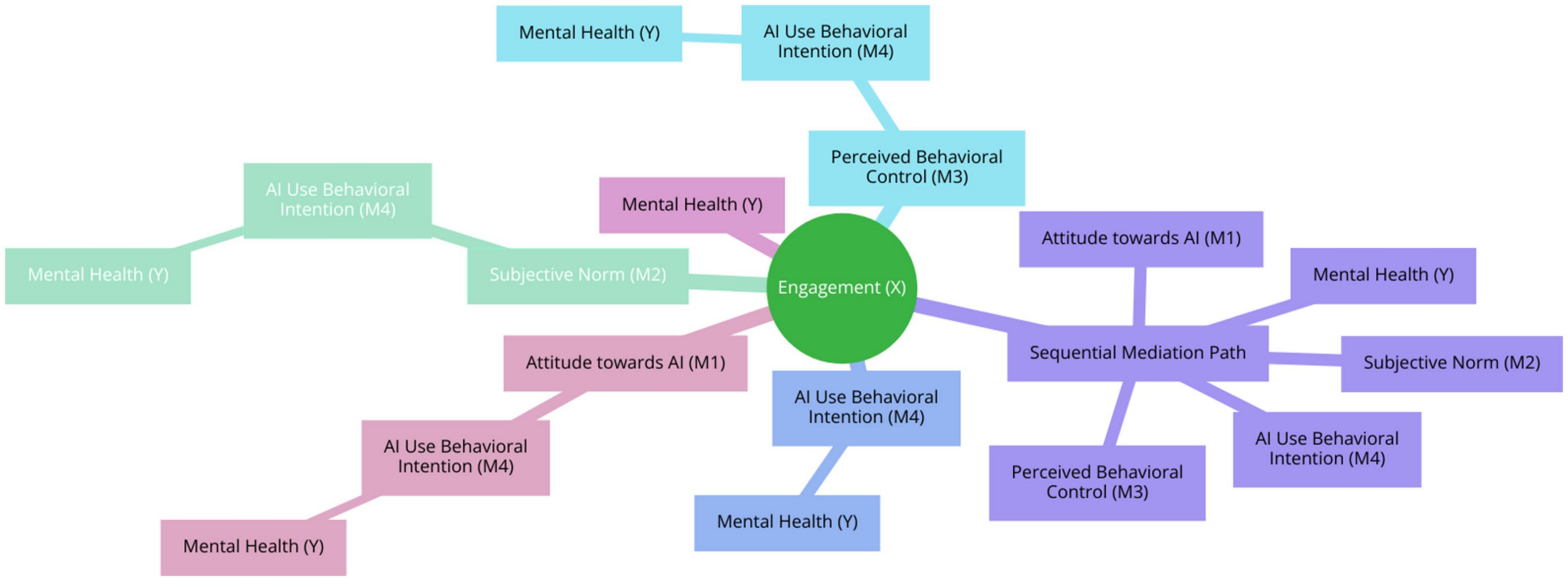
Theoretical model of relationships between the variables.

The following Figure 1 depicts the mediated chain model of relationships between the variables.

Based on the above displayed model, this study proposes the following hypotheses:

*Hypothesis 1*: There is a significant positive relationship between Chinese college students’ engagement (X) and their mental health (Y).

*Hypothesis 2*: Attitude toward AI (M1) mediates the relationship between Chinese college students’ engagement (X) and their mental health (Y).

*Hypothesis 3*: Subjective norm (M2) mediates the relationship between Chinese college students’ engagement (X) and their mental health (Y).

*Hypothesis 4*: Perceived behavioral control over AI use (M3) mediates the relationship between Chinese college students’ engagement (X) and their mental health (Y).

*Hypothesis 5*: AI use behavioral intention (M4) mediates the relationship between Chinese college students’ engagement (X) and their mental health (Y).

*Hypothesis 6*: Chinese college students’ attitude toward AI (M1), subjective norm (M2), and perceived behavioral control over AI use (M3) positively predict their AI use behavioral intention (M4).

*Hypothesis 7*: AI use behavioral intention (M4) mediates the relationship between Chinese college students’ engagement (X) and their mental health (Y).

*Hypothesis 8*: The relationship between Chinese college students’ engagement (X) and their mental health (Y) is sequentially mediated by their attitude toward AI (M1), subjective norm (M2), perceived behavioral control over AI use (M3), and AI use behavioral intention (M4).

**Figure 2:**
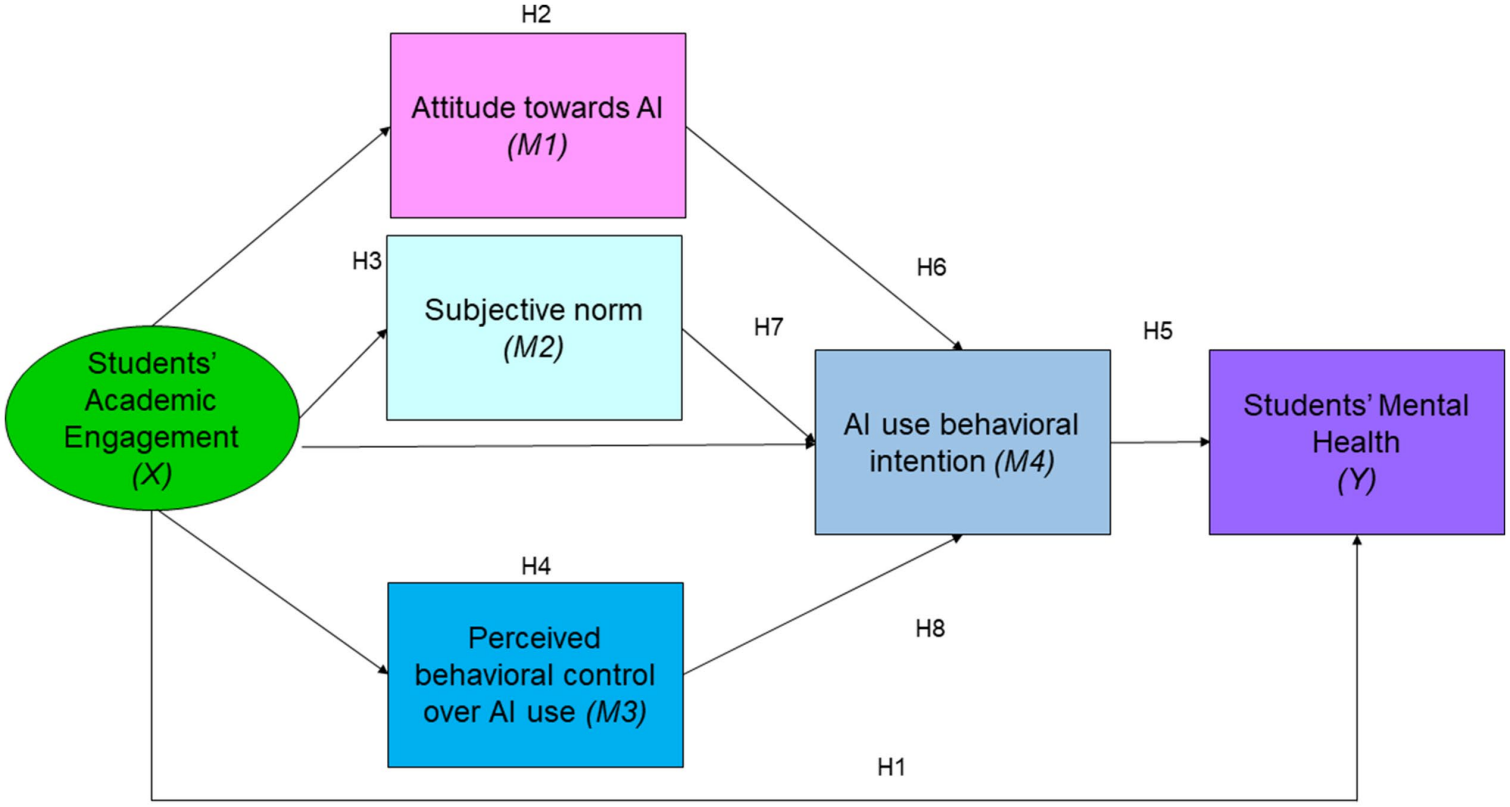
Full set of hypotheses.

This study aims to provide valuable insights into how AI can be leveraged to improve mental health education among Chinese college students. By understanding the factors that influence students’ engagement with AI technologies, educational institutions can develop more effective strategies to enhance mental health support and academic outcomes. The findings can inform policy decisions, promote the adoption of AI in educational settings, and ultimately contribute to the overall well-being of students. The full set of hypotheses is displayed in Figure 2.

## Methods

### Participants

The study involved a total of 2,423 Chinese college students. The ages of the participants ranged from 18 to 23 years, with a mean age of approximately 20.53 years and a standard deviation of 1.51 years. This age distribution reflects the typical age range of undergraduate students in Chinese universities.

Regarding gender distribution, the sample comprised 1,001 male participants and 1,422 female participants. Males constituted 41.3% of the sample, while females made up 58.7%. This indicates a slightly higher representation of female students in the study, which aligns with some existing trends in higher education demographics.

The survey assessed household income to categorize participants into socioeconomic levels. The question was, “What is your household’s annual income (before taxes)?.” The Response Scale was in Chinese Yuan, and it ranged from (1) Less than ¥50,000 to (6) More than ¥500,000. The socioeconomic status of the participants was categorized into three levels. A total of 318 participants (13.1%) were classified as belonging to the low socioeconomic level. The majority of the participants, 2,000 students (82.5%), were classified as middle socioeconomic level. Lastly, 105 participants (4.3%) were from the high socioeconomic level. This distribution highlights that the majority of the students came from middle socioeconomic backgrounds, with fewer participants from low and high socioeconomic levels. Regarding specialties distribution, 30% of the students belonged to STEM (Science, Technology, Engineering, and Mathematics) fields. This includes disciplines such as engineering, technology, information technology, computer science, natural sciences (physics, chemistry, biology), and mathematics. Health sciences accounted for approximately 15% of the student population. This includes medicine, dentistry, nursing, allied health, and pharmacy. Business and economics disciplines represented about 10% of the students. The remaining 45% of the students were distributed among humanities, social sciences, arts, education, and other disciplines. Social sciences, including psychology, sociology, and political science, formed a significant portion of this category.

Overall, the participant demographic provides a diverse representation of Chinese college students, encompassing a range of ages, gender, and socioeconomic backgrounds.

### Procedure

Prior to launching the survey, the study underwent review and approval by the Zhoukou Vocational and Technical College institutional review board. All participants were provided with an informed consent form at the beginning of the survey, which they had to read and agree to before proceeding. The consent form detailed the study’s purpose, procedures, potential risks, benefits, and the voluntary nature of participation. It also assured participants of their right to withdraw from the study at any time without any penalty. Confidentiality and anonymity were strictly maintained; personal identifying information was not collected, and all responses were stored securely in encrypted databases. Only the first and second authors had access to the data, and it was used solely for research purposes, with a commitment that participants’ responses would not be shared with third parties. Upon completion of the study, results were reported in aggregate form to prevent any identification of individual participants.

The development of the present study involved two main steps: dissemination of the study among colleges in China using social networks, and conducting the survey via a website. To effectively disseminate the study, social networks were utilized extensively. Official accounts for the study were created on popular Chinese social media platforms such as WeChat, Weibo, and QQ. These platforms, widely used by college students, ensured broad reach and engagement. The dissemination strategy involved creating an invitation post to explain the study’s purpose, importance, and potential impact. Collaboration with influential education-related accounts and college student organizations helped amplify the message. Regular updates and reminders were posted to maintain interest and encourage participation. Special attention was given to responding to inquiries and comments to build trust and transparency with potential participants using the academic email of the first author.

The survey was conducted through Questionnaire Star, using a secure website designed specifically for the study. The website featured a user-friendly interface to ensure easy navigation and participation. Participants accessed the survey through links shared on social media posts, QR codes, and direct invitations sent through college mailing lists. Upon entering the website, participants were provided with detailed information about the study, including its objectives, procedures, and assurances of anonymity, voluntariness, the option to withdraw, and the commitment that participants’ responses would not be shared with third parties. Clear instructions on how to complete the survey were given, and the estimated time required was communicated upfront. To encourage honest and thoughtful responses, the survey was designed to be concise yet comprehensive, covering all necessary areas of inquiry. The website also included a Frequently Asked Questions (FAQ) section and contact information for technical support.

### Measures

#### Students’ academic engagement

The Utrecht work engagement student (UWES-3) is a scale designed to evaluate work engagement (Schaufeli et al., 2019) but has been previously applied to academic success (Yang, 2024). It included three dimensions: vigor (Studying makes me feel strong and energetic), dedication (I’m passionate about what I’m studying), and absorption (I find it difficult to disengage from my studies). Previous studies reported adequate reliability data, as Cronbach’s Alphas 0.82 (Yang, 2024), and 0.70 (Dominguez-Lara et al., 2021).

#### Students’ attitudes toward AI

To asses this variable, the subscale of Perceived usefulness of AI use from the TAME-ChatGPT scale has been applied (Abdaljaleel et al., 2024), which consists of six items based on a previous study (Sallam et al., 2023). The redaction of the items has been adapted to refer to a more general AI use instead of only ChatGPT use. The English version of the scale was adapted through a back-translation procedure to Chinese, following the recommendations by Kim (2001) regarding that the focus of translation should be on the global meaning of the statements, instead of a textual translation.

#### Subjective norm toward AI use

To asses this variable the subscale of Subjective norm (Li K., 2023) was applied. The original scale includes six items, showing adequate reliability (Li K., 2023) (Cronbach’s Alphas = 0.92) and it was originally designed for AI-based tools use. A Likert 5-point scale was used from 1 (mostly disagree) to 5 (mostly agree).

#### Perceived behavioral control of AI use

The scale of behavioral control with three items has been used (Al-Emran et al., 2021), following the adaptation made by Al-Sharafi et al. (2023).

#### AI use behavioral intention

To asses this variable the subscale of Behavioral intention toward AI-based systems (Li K., 2023) was applied. The original scale includes four items, reaching a reliability level of Cronbach’s Alphas of 0.88 (Li K., 2023).

#### Students’ mental health

To evaluate mental health among college students, the Center for Epidemiologic Studies Depression Scale (CES-D) has been utilized. Originally developed by Radloff (1977), this scale is intended for epidemiological studies to measure the extent of depressive symptoms and identify individuals at risk of depression within the general population. The CES-D is a self-assessment tool comprising 20 items, each rated on a five-point scale from 1 (“rarely or none of the time”) to 5 (“all of the time”). For this research, a modified five-point response scale, adapted from the original four-point Likert scale, was employed to enhance the variance in depressive symptoms among college students, following the methodology suggested by Zhang et al. (2017). The existing literature on the CES-D has identified at least 20 factor solutions across various populations and subpopulations. Notably, the CES-D includes an interpersonal factor, a feature not commonly found in other widely used instruments. The CES-D has shown strong reliability and validity across different Chinese populations, including elderly communities Zhang et al. (2011) and individuals who have attempted suicide (Yang et al., 2015). The full CES-D Chinese version is available at Chin et al. (2015).

### Data analyses

To examine the proposed hypotheses, we utilized the PROCESS macro for SPSS (Version 4.2) developed by Hayes (2022). Specifically, we employed Model 80, a chain mediation model that allows for multiple mediators to be tested sequentially. This model is appropriate for our study as it enables the assessment of direct and indirect effects of students’ engagement (X) on mental health (Y) through a series of mediators: attitude toward AI (M1), subjective norm (M2), perceived behavioral control over AI use (M3), and AI use behavioral intention (M4).

#### Confidence intervals

The lower level confidence interval (LLCI) and upper level confidence interval (ULCI) are used to determine the significance of the effects. A confidence interval that does not include zero indicates a significant effect. For this study, a 95% confidence interval was used: LLCI: The lower bound of the 95% confidence interval. ULCI: The upper bound of the 95% confidence interval. If the LLCI and ULCI for an effect do not straddle zero, the effect is considered statistically significant. This criterion ensures that we can be 95% confident that the true effect size is not zero, thereby confirming the presence of a significant mediation effect.

## Results

### Descriptive statistics and Pearson’s correlation matrix

The descriptive statistics and correlation matrix for the variables under study are presented in Table 1. The results reveal significant relationships between various predictors and the outcome variable, mental health, among Chinese college students.

**Table 1:** Descriptive statistics and correlation matrix.

Variable	Mean	SD	1	2	3	4	5	6
1. Students’ engagement	4.009	0.895	*0.75*					
2. Attitude toward AI	4.236	0.795	0.351**	*0.78*				
3. Subjective norm	3.074	1.145	0.032	0.158**	*0.80*			
4. Perceived behavioral control over AI use	4.044	0.749	0.476**	0.269**	0.118**	*0.79*		
5. AI use behavioral intention	3.807	0.830	0.563**	0.330**	0.092**	0.380**	*0.82*	
6. Mental health	3.685	0.478	0.252**	0.160**	0.031	0.306**	0.264**	*0.76*

**p < 0.01. *N* = 2,423. Values in italics in the diagonal are Cronbach’s Alphas.

First, there is a notable positive correlation between students’ engagement and mental health, indicating that higher levels of engagement are associated with better mental health outcomes. This supports the hypothesis that engagement plays a crucial role in students’ mental well-being.

The mediating variables related to AI also show interesting patterns. Attitude toward AI is significantly and positively correlated with both students’ engagement and mental health. This suggests that a positive attitude toward AI may enhance the benefits of engagement in mental health. Similarly, subjective norms and perceived behavioral control over AI use are positively related to engagement, indicating that social influences and confidence in managing AI contribute to students’ engagement levels.

Further, AI use of behavioral intention is significantly correlated with all other mediators and mental health. This finding supports the notion that intentions to use AI are influenced by attitudes, norms, and perceived control, and these intentions, in turn, impact mental health.

Overall, the results support the hypothesized chain mediation model. Students’ engagement positively affects their mental health, and this relationship is mediated by their attitude toward AI, subjective norm, perceived behavioral control over AI use, and AI use behavioral intention. The sequential nature of these mediators highlights the complex interplay between engagement and mental health, influenced by various factors related to AI use.

### Hypotheses testing

#### Direct effect

The first hypothesis predicted a significant positive relationship between students’ engagement and their mental health (*β* = 0.0575; *p* < 0.05). The analysis confirmed this hypothesis, indicating that higher levels of engagement are associated with better mental health outcomes as Table 2 shows.

**Table 2:** Model summaries.

Outcome variable	*R*	*R* ^2^	MSE	*F*	df1	df2	*p*
Attitude	0.3508	0.1231	0.5540	339.8202	1	2,421	0.0000
Subjective norm	0.0317	0.0010	1.3092	2.4410	1	2,421	0.1183
Perceived behavioral control	0.4760	0.2266	0.4341	709.3696	1	2,421	0.0000
AI use behavioral intention	0.5921	0.3505	0.4481	326.2703	4	2,418	0.0000
Mental health	0.3510	0.1232	0.2004	67.9281	5	2,417	0.0000

#### Indirect effects

Hypothesis 2 posited that attitude toward AI would mediate the relationship between students’ engagement and their mental health. The analysis showed that the indirect effect through attitude toward AI was not significant (*β* = 0.0135), indicating that this mediation was not supported, despite its marginal statistical value. Hypothesis 3 suggested that subjective norm would mediate the relationship between students’ engagement and their mental health. The results indicated that the indirect effect through subjective norm was also not significant (*β* = −0.0005), failing to support this hypothesis. The fourth hypothesis proposed that perceived behavioral control over AI use would mediate the relationship between students’ engagement and their mental health. The analysis confirmed a significant indirect effect through perceived behavioral control over AI use (*β* = 0.1039; *p* < 0.05), supporting Hypothesis 4. Hypothesis 5 predicted that AI use behavioral intention would mediate the relationship between students’ engagement and their mental health. The analysis supported this hypothesis (β = 0.0063; *p* < 0.05), showing a significant indirect effect through AI use behavioral intention. Table 3 shows the coefficients for the Regression models.

**Table 3:** Coefficients.

Predictor	Coeff	SE	*t*	*p*	LLCI	ULCI	β
**Outcome variable:** Attitude							
Students’ engagement	0.3115	0.0169	18.434	0.0000	0.2784	0.3447	0.3508
**Outcome variable:** Subjective norm							
Students’ engagement	0.0406	0.0260	1.5624	0.1183	−0.0104	0.0915	0.0317
**Outcome variable:** Perceived behavioral control							
Students’ engagement	0.3984	0.0150	26.634	0.0000	0.3691	0.4278	0.4760
**Outcome variable:** AI use behavioral intention							
Students’ engagement	0.4242	0.0180	23.628	0.0000	0.3890	0.4594	0.4574
Attitude toward AI	0.1361	0.0186	7.3078	0.0000	0.0995	0.1726	0.1303
Subjective norm	0.0306	0.0121	2.5270	0.0116	0.0068	0.0543	0.0422
Perceived behavioral control	0.1352	0.0209	6.4668	0.0000	0.0942	0.1763	0.1221
**Outcome variable:** Mental health							
Students’ engagement	0.0307	0.0133	2.3026	0.0214	0.0046	0.0568	0.0575
Attitude toward AI	0.0232	0.0126	1.8418	0.0656	−0.0015	0.0479	0.0386
Subjective norm	−0.0064	0.0081	−0.7958	0.4262	−0.0223	0.0094	−0.0155
Perceived behavioral control	0.1392	0.0141	9.8656	0.0000	0.1115	0.1668	0.2183
AI use behavioral intention	0.0789	0.0136	5.8045	0.0000	0.0523	0.1056	0.1372

#### Chain mediational effects

**Figure 3:**
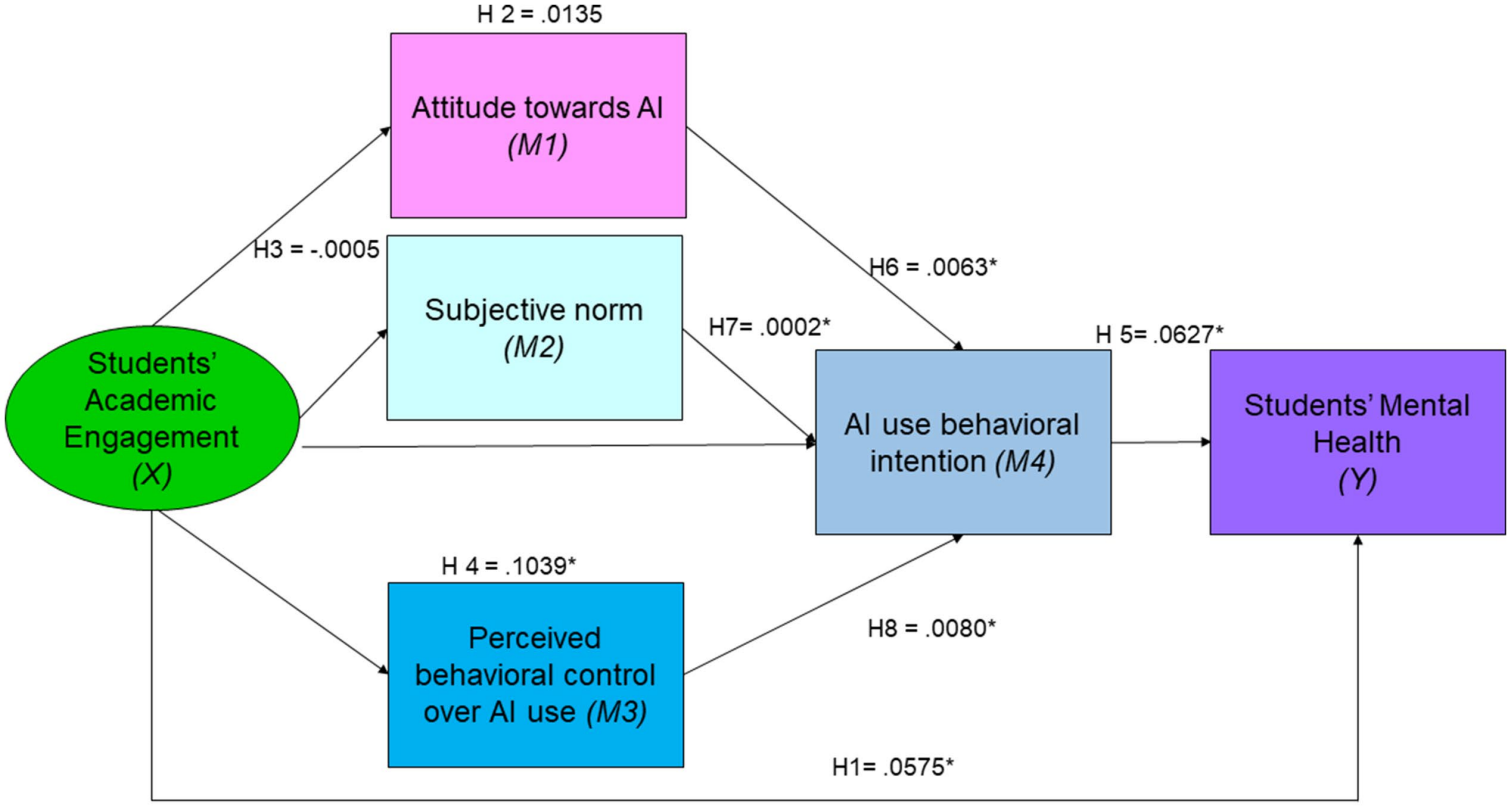
Standardized estimates for the direct, indirect and chain mediating effects.

Hypothesis 6 proposed that Chinese college students’ attitude toward AI (β = 0.1303; *p* < 0.001), subjective norm (β = 0.0422; *p <* 0.01), and perceived behavioral control over AI use (β = 0.1221; *p* < 0.001) would positively predict their AI use behavioral intention. The results supported this hypothesis, indicating that all three factors significantly predicted AI use behavioral intention. Hypothesis 7 suggested that AI use behavioral intention would mediate the relationship between students’ engagement and their mental health. The analysis confirmed this hypothesis (β = 0.0627; *p* < 0.05), showing that AI use behavioral intention significantly mediated the relationship. Hypothesis 8 posited that the relationship between students’ engagement and their mental health would be sequentially mediated by their attitude toward AI (β = 0.0063; *p* < 0.05), subjective norm (β = 0.0002; *p* < 0.05), perceived behavioral control over AI use (β = 0.0080; *p* < 0.05), and AI use behavioral intention. The results supported this hypothesis, demonstrating a significant sequential mediation effect, as Table 4 displays, as well as Figure 3 represents.

**Table 4:** Total, direct, and indirect effects.

Effect type	Effect	SE	LLCI	ULCI	β
Total effect (*t* = 12.790; *p* < 0.001)	0.1343	0.0105	0.1137	0.1549	0.2516
Direct effect (*t* = 2.302; *p* < 0.05)	0.0307	0.0133	0.0046	0.0568	0.0575
**Indirect effects**					
Total indirect effect	0.1036	0.0106	0.0835	0.1245	0.1941
Students → Attitude → Mental health	0.0072	0.0044	−0.0012	0.0161	0.0135
Students → Subjective norm → Mental health	−0.0003	0.0004	−0.0013	0.0005	−0.0005
Students → Perceived behavioral control → Mental health	0.0555	0.0067	0.0423	0.0688	0.1039
Students → AI use behavioral intention → Mental health	0.0335	0.0062	0.0212	0.0457	0.0627
Students → Attitude → AI use behavioral intention → Mental health	0.0033	0.0008	0.0019	0.0051	0.0063
Students → Subjective norm → AI use behavioral intention → Mental health	0.0001	0.0001	0.0000	0.0003	0.0002
Students → Perceived behavioral control → AI use behavioral intention → Mental health	0.0043	0.0011	0.0023	0.0064	0.0080

## Discussion

The present study was aimed to test a TPB-based model suggesting that Academic engagement –mental health relationships was serially mediated by students’ attitudes toward AI use, subjective norm, perceived behavioral control and Students’ AI use behavioral intention, which in term influences on mental health among Chinese college students.

The first hypothesis, which predicted a significant positive relationship between students’ engagement and their mental health, was confirmed by the analysis. This finding aligns with existing literature that highlights the importance of academic engagement in promoting students’ mental well-being. For example, Finn and Zimmer (2012) found that higher levels of academic engagement are associated with better mental health outcomes, including reduced anxiety and depression. Similarly, Dumford and Miller (2018) reported that students who are more engaged in their academic pursuits tend to have higher levels of psychological well-being.

However, this finding contrasts with some previous studies that have suggested that the pressure to perform academically can lead to increased stress and mental health issues (Conley et al., 2015; Conley et al., 2017). These studies argue that while engagement can have positive effects, the high expectations and workload associated with academic involvement can also contribute to negative mental health outcomes. Thus, the relationship between engagement and mental health may be more complex than previously thought, necessitating a balanced approach that promotes engagement while also addressing potential stressors.

The second hypothesis posited that attitude toward AI would mediate the relationship between students’ engagement and their mental health. However, the analysis did not find a significant indirect effect through attitude toward AI. This result suggests that while students’ attitudes toward AI are important, they may not play a pivotal role in linking engagement to mental health. This finding is consistent with the other works (Pan, 2020; Zaineldeen et al., 2020), which indicate that attitudes toward technology, while influential, are often overshadowed by more immediate contextual factors such as usability and perceived usefulness. This evidence is in line with a large amount of research showing the predictive power of attitudinal measures on wellbeing (Mason Stephens et al., 2023; Sibley et al., 2020).

Similarly, the findings did not support that subjective norms would mediate the relationship between students’ engagement and their mental health. This result contrasts with studies that emphasize the role of social influence in technology adoption (Venkatesh and Davis, 2000). While subjective norms can affect technology use intentions, their impact on the specific context of mental health outcomes appears limited, highlighting the need for further investigation into other mediating factors. This contrasting evidence can be connected with the TPB-based research about cheating behavior in the academic context (Stone et al., 2010). Given that the use of AI in the classroom, specifically to complete exercises and academic tasks, can be viewed as misconduct, subjective norms can be ambivalent about this behavior. On the one hand, it can be considered a technological advancement and a demonstration of technical capability (Norzelan et al., 2024), but on the other, it can be considered academic misconduct that should be avoided and punished (Maulana, 2019). In this sense, modified versions of the TPB, exploring the justification of this behavior, could clarify the fault of the significance of subjective norm in the present study (Scrimpshire et al., 2017).

The fourth hypothesis, which proposed that perceived behavioral control over AI use would mediate the relationship between students’ engagement and their mental health, was supported. This finding aligns with the Theory of Planned Behavior (Ajzen, 1991), which posits that perceived control is a critical determinant of behavior. Several studies support this notion, particularly in the context of educational technology. For instance, Alalwan et al. (2018) demonstrated that students who feel confident in their ability to use technology, including AI, effectively are more likely to experience positive mental health outcomes, as they can better leverage these tools to enhance their learning and well-being.

However, it is important to recognize that this relationship is not always straightforward. Research by Nazari et al. (2021) highlights that for some students, increased access to AI tools can introduce stress, particularly when they feel overly reliant on the technology or lack full mastery of its use. In such cases, perceived behavioral control may not always lead to positive mental health outcomes, especially if students experience anxiety about their ability to keep up with the demands of using AI. Furthermore, a study by Hsia (2016) emphasizes that perceived behavioral control is a significant predictor of positive outcomes in various digital learning environments. Students who exhibit high levels of perceived control tend to engage more effectively in self-regulated learning, which not only improves academic performance but also contributes to better mental health. In this context, perceived control over AI use can serve as a buffer against stress, enabling students to navigate challenges more effectively.

Thus, while the findings of this study align with the Theory of Planned Behavior and are supported by several studies, it is crucial to consider that the effects of perceived behavioral control over AI use may vary across different student populations. Factors such as digital literacy, confidence in technology use, and individual differences in stress response should be considered in future research to provide a more comprehensive understanding of how AI impacts students’ mental health.

Hypothesis five predicted that AI use behavioral intention would mediate the relationship between students’ engagement and their mental health, and the analysis confirmed this hypothesis. This result supports the idea that behavioral intentions are strong predictors of actual behavior and subsequent outcomes (Sheeran, 2002). When students intend to use AI tools, they are more likely to engage with these technologies in ways that benefit their mental health. This is consistent with findings from Venkatesh et al. (2012), where behavioral intentions regarding technology use have been shown to significantly influence actual engagement with technological tools, ultimately leading to positive educational and psychological outcomes.

Moreover, Hagger et al. (2018) highlight that the translation of intentions into behavior is often facilitated when there is sufficient perceived control, further reinforcing the relationship between AI use intentions and student engagement. In the context of technology adoption, this has been observed in several areas, such as e-learning platforms, mobile applications for educational purposes, and AI-driven tools in education. For instance, in e-learning environments, students’ perceived ease of use and control over the technology significantly impact their intention to engage with online learning tools. Similarly, the adoption of AI-driven platforms like adaptive learning systems, which tailor content based on individual student needs, is strongly influenced by students’ perceived control and confidence in using such technologies (Alqahtani et al., 2023). Therefore, this mediation effect of behavioral intention is robust across various technology contexts, especially in educational technologies where user engagement depends on both the perceived utility of the tool and the user’s ability to navigate it effectively.

Hypothesis six proposed that Chinese college students’ attitude toward AI, subjective norms, and perceived behavioral control over AI use would positively predict their AI use behavioral intention. The analysis confirmed this hypothesis, indicating that these factors collectively shape students’ intentions to use AI. This finding aligns with the Unified Theory of Acceptance and Use of Technology (Venkatesh et al., 2003), which emphasizes the combined influence of attitudes, social norms, and perceived control on technology use intentions.

The predictive power of attitudes, subjective norms, and perceived control on behavioral intentions has been well-documented across several domains. For example, Armitage and Conner (2001), in their meta-analysis, demonstrated that attitudes, followed by subjective norms and perceived control, consistently predicted behavioral intentions in various behavioral contexts, including health-related behaviors. Similarly, Cooke et al. (2016), in their systematic review, confirmed that these constructs strongly influence behavioral intentions, reinforcing their role in shaping actions within technology adoption contexts.

Furthermore, Kumar and Nayak (2023) provided empirical support for the Theory of Planned Behavior, showing that attitudes play a crucial role in influencing behavioral intention, particularly in educational technology. They found that positive attitudes toward AI significantly drive students’ intentions to engage with AI, while subjective norms (influences from peers or faculty) and perceived behavioral control (students’ confidence in using AI) further reinforce this intention. Thus, these findings align with both the Unified Theory of Acceptance and Use of Technology and the broader literature on the Theory of Planned Behavior, reinforcing the notion that attitude is a crucial antecedent of behavioral intention, followed by subjective norms and perceived behavioral control.

Hypothesis seven suggested that AI use of behavioral intention would mediate the relationship between students’ engagement and their mental health. The analysis supported this hypothesis, demonstrating that students who intend to use AI tools are more likely to see improvements in their mental health through increased engagement. This result highlights the importance of fostering strong behavioral intentions to use AI as a pathway to better mental health outcomes. As AI use can be viewed as a resource to deal with highly demanding tasks, behavioral intentions to use them could reduce depression and anxiety associated to academic exhaustion (Li and Anila, 2023).

Finally, hypothesis eight posited that the relationship between students’ engagement and their mental health would be sequentially mediated by their attitude toward AI, subjective norm, perceived behavioral control over AI use, and AI use behavioral intention. The analysis confirmed this complex mediation chain, suggesting that these factors work together in a sequential process to influence mental health outcomes. This finding underscores the interconnected nature of psychological constructs and their collective impact on behavior and well-being.

To sum up, one of the key strengths of this research is its comprehensive approach, examining multiple psychological constructs—attitude, subjective norm, perceived behavioral control, and behavioral intention—in a sequential mediation model. This approach provides a deeper understanding of how these variables interact to influence students’ mental health, moving beyond simple relationships to explore the complexity of behavioral change. By capturing the progression of attitudes, social influences, and self-efficacy beliefs, the study highlights the indirect pathways that link AI engagement with mental health, emphasizing the importance of fostering positive attitudes and enhancing perceived control.

Additionally, the research contributes to the literature by extending the Theory of Planned Behavior (TPB) and Unified Theory of Acceptance and Use of Technology (UTAUT) to AI use in education, demonstrating their relevance in predicting both behavioral and mental health outcomes. The focus on a large sample of Chinese college students enhances the generalizability of the findings, offering timely insights for higher education contexts where AI adoption is rapidly increasing. Methodologically, the use of structural equation modeling (SEM) adds rigor, ensuring that the complex mediation process is empirically validated and the study’s internal validity is strengthened by controlling for confounding variables.

### Limitations of the present study

While the study provided valuable insights into the demographics and opinions of Chinese college students, several limitations should be acknowledged.

First, the sample consisted solely of students from Chinese colleges, which may limit the generalizability of the findings to other populations or educational systems. The demographic characteristics, such as age and socioeconomic status, were representative of the specific context of Chinese higher education, but may not reflect the diversity found in other countries or regions. Cultural values and practices can affect the relationships between predictors and criteria (Kumar and Nayak, 2023).

Second, the reliance on self-reported data through an online survey introduces potential biases. Participants might have provided socially desirable responses or might not have fully understood some of the questions, despite efforts to make the survey concise and comprehensive. Additionally, the use of an online platform may have excluded students with limited internet access or those who are less comfortable with digital tools, potentially skewing the sample toward more technologically savvy individuals (Lau and Carney, 2024). Moreover, potential factors that are not included in this research could affect the findings, as current mental health status, knowledge of mental health, parental support for health problems among others.

Third, while social networks were an effective tool for disseminating the study, this method might have introduced selection bias. Students who are more active on social media and more engaged with online communities were more likely to participate, which might not represent the broader student population.

Fourth, the cross-sectional nature of the study provides a snapshot of the participants’ views and characteristics at a single point in time. This design does not allow for analysis of changes over time or the identification of causal relationships. Longitudinal studies would be beneficial to understand how students’ opinions and demographics evolve (Li F. et al., 2022).

Finally, the confidentiality and anonymity of the survey were maintained, but the assurance of these factors might not have been perceived equally by all participants. Concerns about data privacy, despite reassurances, could have influenced the honesty of the responses (Bowden et al., 2021).

These limitations suggest areas for future research, such as expanding the study to include a more diverse sample, employing mixed methods to complement self-reported data, and conducting longitudinal analyses to track changes over time. Despite these limitations, the study offers significant contributions to understanding the demographics and views of Chinese college students.

### Suggestions for educators and higher education institutions

Given the positive link between academic engagement and mental health, educators and institutions should focus on enhancing student engagement through interactive teaching methods that encourage active learning (Nguyen et al., 2024). Incorporating group projects, discussions, and problem-solving can deepen student involvement. Connecting coursework to real-world applications also boosts motivation (Fazil et al., 2024).

To utilize AI in mental health education, institutions should invest in AI-driven tools that offer personalized support and interventions (Rajaei, 2024). These tools can identify at-risk students and provide tailored resources. Educators must be trained to integrate AI technologies, such as applications that monitor engagement and provide real-time feedback (Li Z., 2023).

The study found that students’ attitudes toward AI affect their engagement and mental health. To encourage positive attitudes, institutions should provide clear information on AI’s benefits and limitations (Pataranutaporn et al., 2021). Workshops, seminars, and training can help clarify AI and address misconceptions (Delcker et al., 2024). Showcasing case studies and offering hands-on experience with AI tools can further build students’ confidence (Zou et al., 2023).

Subjective norms, or social pressure to use AI, significantly influence technology adoption. Institutions can promote AI by fostering a supportive culture, highlighting endorsements from respected faculty and student leaders (Li X. et al., 2022), and implementing peer mentoring programs where experienced students assist newcomers (Kumar et al., 2020).

To enhance students’ perceived control over AI use, institutions should offer comprehensive training, including tutorials, user guides, and troubleshooting support. A user-friendly environment that facilitates easy access to AI tools is essential (Chai et al., 2022). Integrating AI training into the curriculum ensures that all students acquire the necessary skills and confidence to use these technologies effectively (Ivanov et al., 2024).

Strengthening students’ intention to use AI can be achieved by demonstrating its benefits in improving academic and mental health outcomes. Institutions should emphasize how AI streamlines learning, provides personalized support, and boosts academic performance (Chai et al., 2021). Encouraging goal-setting for AI use can further promote proactive adoption.

Higher education institutions should establish comprehensive policies to ensure the ethical and effective use of AI in education (Chan, 2023), as well as sustained support from the administration for both teachers and students (Rowe and Walinga, 2024). These policies must address data privacy, security, and the ethical implications of AI use (Slimi and Carballido, 2023). Clear guidelines on AI data collection, storage, and usage are necessary to protect students’ privacy and rights (Ghimire and Edwards, 2024), and policies should be regularly updated to reflect technological advancements.

Institutions should also implement mechanisms for continuous feedback to improve AI tools and educational strategies (Chen et al., 2020). Input from students and faculty will help refine these tools and ensure their effectiveness. Regular updates based on feedback will enhance both learning outcomes and mental health support (Maghsudi et al., 2021). These strategies can strengthen academic engagement, integrate AI in mental health education, and foster a supportive environment for Chinese college students.

## Conclusion

This study provides valuable insights into the mechanisms through which academic engagement influences mental health among Chinese college students, particularly through the lens of AI use. By confirming the direct positive relationship between engagement and mental health, as well as identifying key mediating factors such as perceived behavioral control and AI use behavioral intention, the findings highlight the potential of AI tools in enhancing students’ well-being. However, the non-significant mediating effects of attitude toward AI and subjective norm suggest that more research is needed to fully understand the nuances of these relationships. Overall, the study contributes to the growing body of literature on the role of AI in education and offers practical implications for improving mental health support in academic settings.

## Data Availability

The raw data supporting the conclusions of this article will be made available by the authors, without undue reservation.
